# Hyperbaric oxygen protects against myocardial reperfusion injury via the inhibition of inflammation and the modulation of autophagy

**DOI:** 10.18632/oncotarget.22869

**Published:** 2017-12-04

**Authors:** Chunxia Chen, Wan Chen, Yaoxuan Li, Yanling Dong, Xiaoming Teng, Zhihuan Nong, Xiaorong Pan, Liwen Lv, Ying Gao, Guangwei Wu

**Affiliations:** ^1^ Department of Hyperbaric Oxygen, The People’s Hospital of Guangxi Zhuang Autonomous Region, Nanning, Guangxi 530021, P. R. China; ^2^ Department of Emergency, The People’s Hospital of Guangxi Zhuang Autonomous Region, Nanning, Guangxi 530021, P. R. China; ^3^ Department of Neurology, The People’s Hospital of Guangxi Zhuang Autonomous Region, Nanning, Guangxi 530021, P. R. China; ^4^ Department of Pharmacology, Guangxi Institute of Chinese Medicine and Pharmaceutical Science, Nanning, Guangxi 530022, P. R. China; ^5^ Department of Biology and Tennessee Center for Botanical Medicine Research, Middle Tennessee State University, Murfreesboro, TN 37132, USA; ^6^ Department of Cardiology, The People’s Hospital of Guangxi Zhuang Autonomous Region, Nanning, Guangxi 530021, P. R. China

**Keywords:** hyperbaric oxygen, inflammation, autophagy, mammalian target of rapamycin

## Abstract

Our previous study demonstrated that hyperbaric oxygen (HBO) preconditioning protected against myocardial ischemia reperfusion injury (MIRI) and improved myocardial infarction. However, HBO’s effect on MIRI-induced inflammation and autophagy remains unclear. In this study, we investigate the potential impact and underlying mechanism of HBO preconditioning on an MIRI-induced inflammatory response and autophagy using a ligation of the left anterior descending (LAD) coronary artery rat model. Our results showed that HBO restored myocardial enzyme levels and decreased the apoptosis of cardiomyocytes, which were induced by MIRI. Moreover, HBO significantly suppressed MIRI-induced inflammatory cytokines. This effect was associated with the inhibition of the TLR4-nuclear factor kappa-B (NF-κB) pathway. Interestingly, lower expression levels of microtubule-associated protein 1 light chain 3B (LC3B) and Beclin-1 were observed in the HBO-treatment group. Furthermore, we observed that HBO reduced excessive autophagy by activating the mammalian target of the rapamycin (mTOR) pathway, as evidenced by higher expression levels of threonine protein kinase (Akt) and phosphorylated-mTOR. In conclusion, HBO protected cardiomocytes during MIRI by attenuating inflammation and autophagy. Our results provide a new mechanistic insight into the cardioprotective role of HBO against MIRI.

## INTRODUCTION

According to data from 2016, there were approximately 660,000 new myocardial infarction (MI) cases and 305,000 recurrent attacks [1]. These epidemiological data have attracted considerable public concern and point to a considerable health threat for humans. Currently, reperfusion, including percutaneous coronary intervention and thrombolysis, is the most effective therapy to protect ischemic damage during MI. However, the development of therapies to reduce myocardial ischemia reperfusion injury (MIRI) has been disappointing [2]. Finding ways to limit MIRI and effective reperfusion strategies has always been a problem.

Autophagy is a critical factor in the heart during MIRI [3–5]. It is an evolutionarily conserved lysosome-dependent degradation process that plays an essential role in cellular homoeostasis and maintaining instances of nutrient starvation [6, 7]. Current research reports that autophagy plays a dual role in MIRI, involving a slight induction during ischemia to promote cell survival and a sharp increase during reperfusion, triggering myocardial cell death [8, 9]. Abundant evidence supports autophagy-induced cell death during MIRI, notably the appearance of autophagic vacuoles and the recruitment of microtubule-associated protein 1 light chain 3 (LC3) to autophagosomes [10–12]. LC3 proteins are stable and persistent and are widely used to monitor autophagy [13]. Threonine protein kinase (Akt), also known as serine, is a multifunctional adaptor protein that serves several signaling pathways, including the mTOR-mediated autophagy process, nuclear factor kappa-B (NF-κB), and apoptosis [14]. It is widely acknowledged that the Akt-mammalian target of rapamycin (Akt-mTOR) pathway and Beclin1-mediated autophagy/apoptosis pathway are two classical autophagy feedback signaling pathways [15–17]. Therefore, it is of increasing interest to restore impaired autophagy by these two pathways to alleviate MIRI.

Reperfused cardiomyocytes also trigger excessive inflammatory responses and provoke further myocardial damage, which is characterized by a rapid release of cytokines, such as IL-1β, IL-10 and TNF-α [18]. Moreover, TLR4 expression, within the myocardium, significantly increases after MIRI [19]. Importantly, TLR4 activates the downstream transcription factor NF-κB, which leads to the overexpression of pro-inflammatory cytokines and the activation of the apoptotic cascade, as well as the autophagic response [20, 21]. The transcription factor NF-κB may modulate oxidative stress, inflammation, apoptosis and autophagy during MIRI. Thus, NF-κB, through TLR4-mediated signaling, may represent an important therapeutic target in ischemic heart disease.

Hyperbaric oxygen therapy (HBO) is a treatment that involves breathing 100 % O_2_ inside a pressurized chamber (the pressure is higher than 1 absolute atmosphere). HBO has a therapeutic effect on various ischemic diseases in the clinic, especially such diseases as stroke and myocardial infarction. However, most of these diseases are not approved by the U.S Food and Drug Administration as an indication [22]. Thus, an investigation of the underlying mechanism of HBO is urgently needed. Our previous data demonstrated that HBO, combined with madopar, had a neuroprotective effect on 6-hydroxydopamine-induced Parkinson’s rats by reducing oxidative stress and apoptosis. HBO also suppressed inflammation in D-galoctose-induced aging mice by modulating the NF-κB pathway, as well as inhibiting the expression of inflammatory cytokines, such as TNF-α and IL-6 [23, 24]. Recently, we found that HBO has a cardioprotective effect in an MIRI rat model by reducing oxygen stress, improving endothelial function and inhibiting cell apoptosis [25]. However, the mechanism of HBO on MIRI-induced inflammation and autophagy remains unclear.

In this study, we investigated the anti-inflammation and modulated autophagy efficacy of HBO using an classical MIRI model, which are established by ligation of left anterior descending (LAD) coronary artery [11]. We determined the effects of HBO on inflammatory cytokines via the TLR4- NF-κB pathway, as well as the modulation of autophagy via the mTOR pathway. Our study provides a possible mechanistic explanation for the protective effect of HBO against MIRI and provides a rationale for HBO use in the clinical treatment of early reperfusion after an acute myocardial infarction.

## RESULTS

### HBO reduced serum myocardial enzyme levels after MIRI

**Figure 1:**
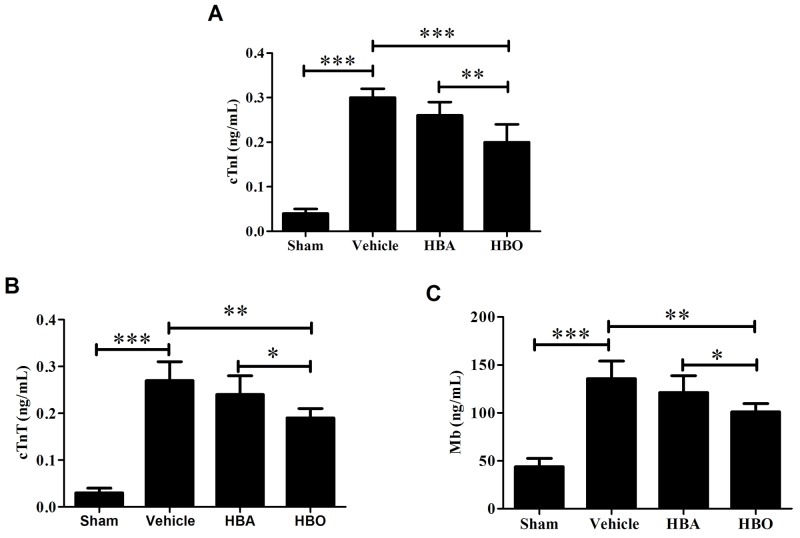
Effect of HBO pretreatment on serum myocardial enzyme activities **(A, B and C)** in MIRI rats. The results are presented as the mean ± standard deviation (n = 7). *P < 0.05; **P < 0.01; ***P < 0.001.

To evaluate the protective effect of HBO against MIRI, we measured the levels of cardiac troponin I (cTnI), cardiac troponin T (cTnT) and myoglbin (Mb) in serum at 60 min after reperfusion. As indicated in Figure 1A, in the vehicle group, the activity of cTnI in serum increased almost 7.5-fold by the end of reperfusion compared with the sham group. Treatment with hyperbaric air (HBA) or HBO reduced the activity of cTnI compared to the vehicle group by 13.33% and 33.33%, respectively. Likewise, the activity of cTnT underwent a sharp increase in the vehicle group in response to the sham group, while this increase was attenuated by the HBO treatment (Figure 1B). The serum activity of Mb in the vehicle group was almost 2.1-fold higher than in the sham group. The pre-administration of HBO significantly decreased Mb by 25.57% compared to the vehicle group (Figure 1C). However, the activities of cTnT or Mb in the HBA group and the vehicle group did not differ significantly (*P* > 0.05).

### HBO reduced inflammatory cytokines levels after MIRI

**Figure 2:**
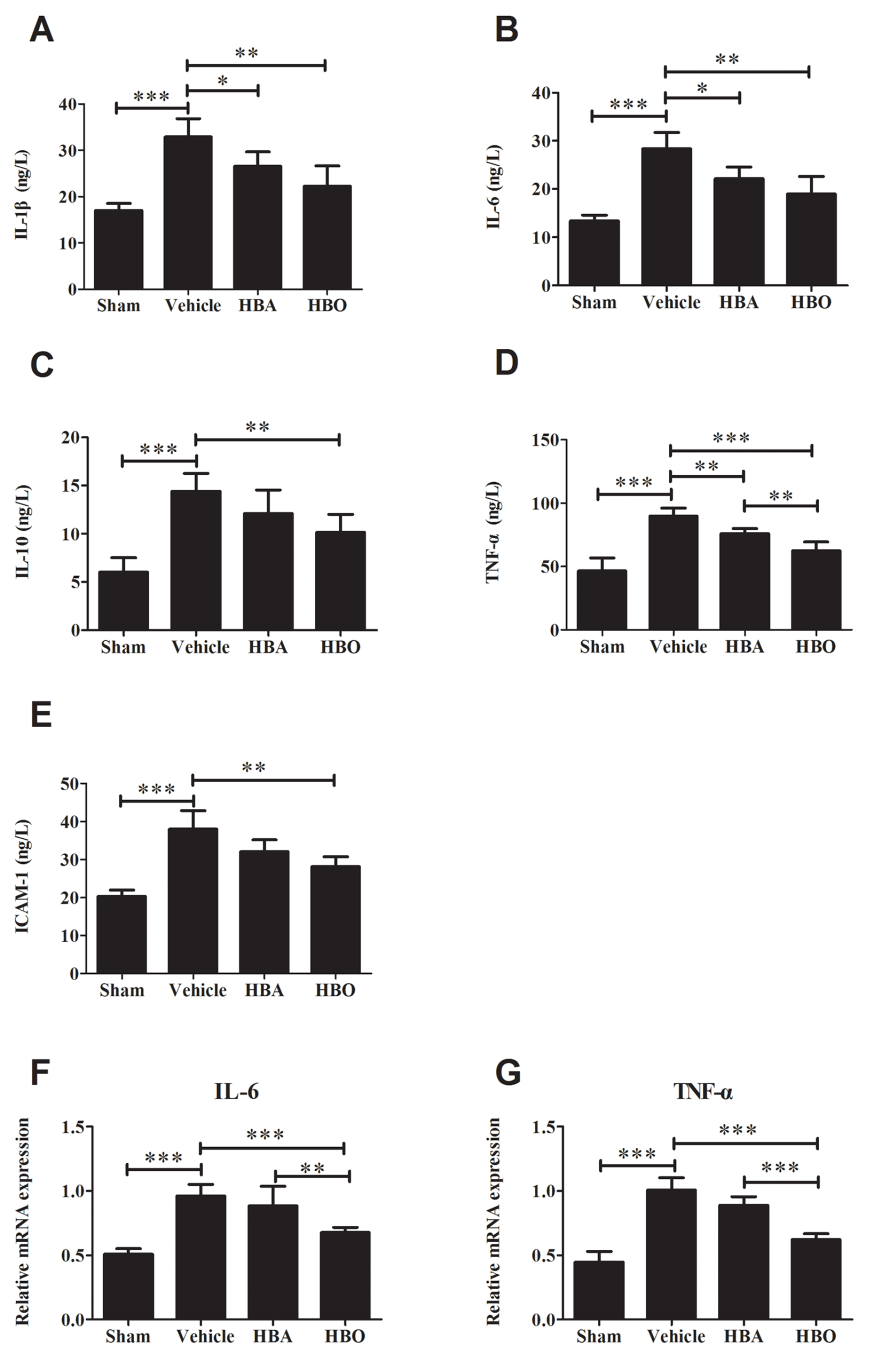
Analysis of inflammatory cytokines levels in HBO-treated MIRI rats **(A, B, C, D, and E)** ELISA assay of IL-1β, IL-6, IL-10, TNF-α, and ICAM-1 levels, respectively. **(F and G)** The gene copies of IL-6 and TNF-α were determined by using RT-PCR. The results are presented as the mean ± standard deviation (n = 8). ^*^P < 0.05; ^**^P < 0.01; ^***^P < 0.001.

Next, to determine whether the HBO treatment alleviates the inflammatory reaction, the inflammatory cytokines, including IL-1β, IL-6, IL-10, TNF-α, and ICAM-1, were measured by ELISA. As shown in Figure 2A, 2B, 2C, 2D, and 2E, a significantly higher expression of IL-1β, IL-6, IL-10, TNF-α, and ICAM-1 was observed in the vehicle group compared to the sham group. However, these changes were reversed in the HBO treatment group. Treatment with HBA produced a trend of decreased inflammatory cytokines. However, the change was not significantly different. Meanwhile, the Real-Time PCR (RT-PCR) analysis (Figure 2F and 2G) revealed that the expressions of IL-6 and TNF-α mRNA in the cardiomyocytes were up-regulated in the vehicle group compared with the sham group. However, the number of gene copies of IL-6 and TNF-α was significantly reduced by the HBO treatment.

### HBO inhibits the expressions of NF-κB, p-IκBα and TLR4 in MIRI rats

**Figure 3:**
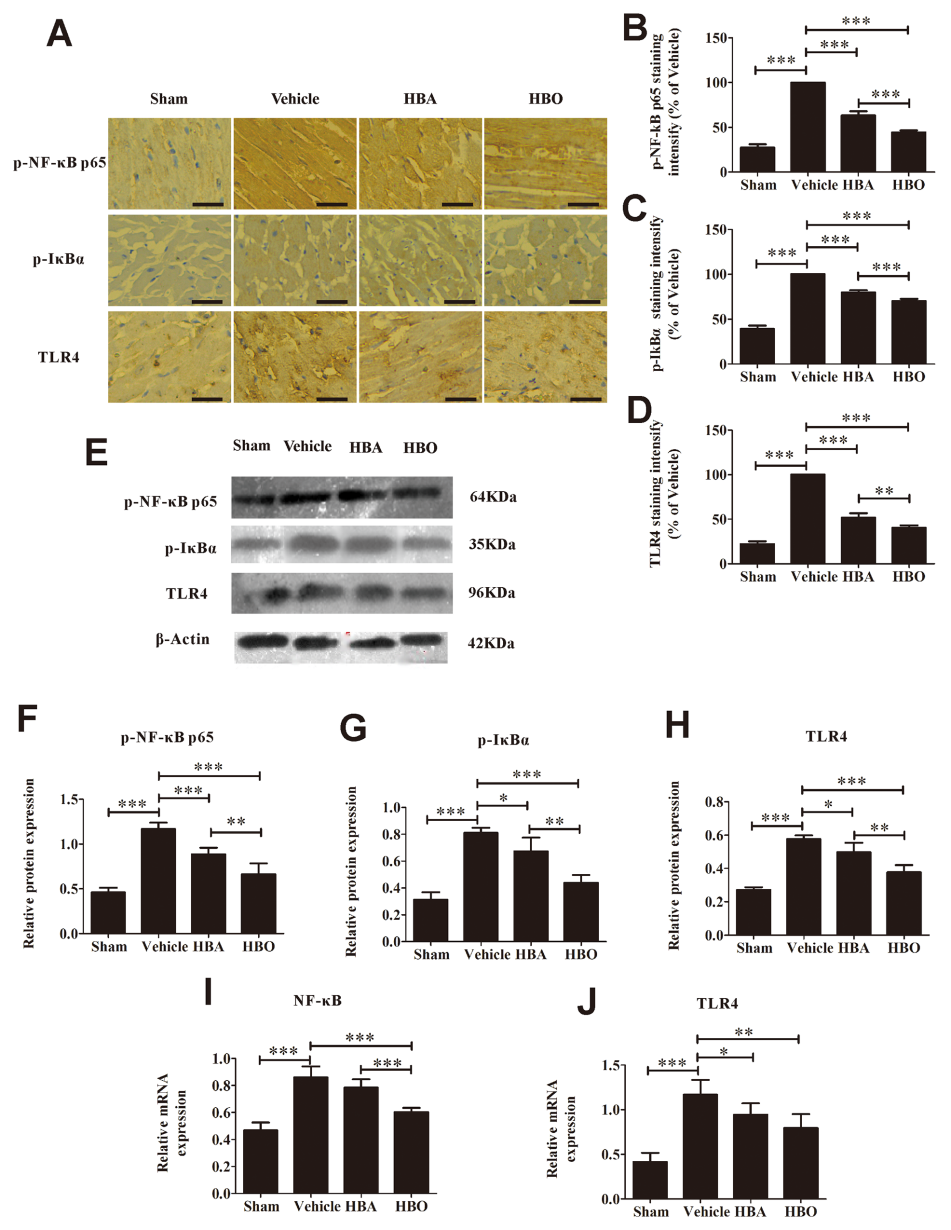
HBO suppresses the activation of TLR4-NF-κB pathway **(A)** Immunohistochemical staining of p-NF-κB p65, p-IκBα, and TLR4 in heart tissue of each group. The scale bar represents 50 μm. **(B, C and D)** Quantitative densitometric analysis of p-NF-κB p65, p-IκBα, and TLR4 staining slides of rats subjected to MIRI. Data were normalized with vehicle and presented as percentage rates. (E) Representative images of the Western blot. **(F, G and H)** Quantitative analysis of p-NF-κB p65, p-IκBα, and TLR4 protein expression by Western blot with β-Actin as an internal standard. **(I and J)** The gene copies of NF-κB and TLR4 were determined by using RT-PCR. The results are presented as the mean ± standard deviation (n = 5).^*^P < 0.05; ^**^P < 0.01; ^***^P < 0.001.

To clarify how HBO reduces MIRI, we detected TLR4-NF-κB signaling molecules by immunohistochemistry, western blotting and PCR. The protein expression of p-NF-κB p65 and p-IκBα in the myocardial tissues was much higher in the vehicle group than in the sham group and was significantly decreased by the HBO treatment (Figure 3A, 3B, 3C, 3E, 3F, and 3G). Interestingly, the overexpression of NF-κB and TLR4 mRNA was observed in the myocardial tissues of the MIRI rats compared with a low expression in the sham group. In contrast, the HBO treatment significantly decreased the expression of NF-κB and TLR4 mRNA (Figure 3I and 3J). As expected, the protein expression of TLR4 in the myocardial tissues was significantly decreased by the HBO group compared with the vehicle group (Figure 3A, 3D, 3E, 3H, and 3J).

### HBO inhibited myocardium apoptosis

**Figure 4:**
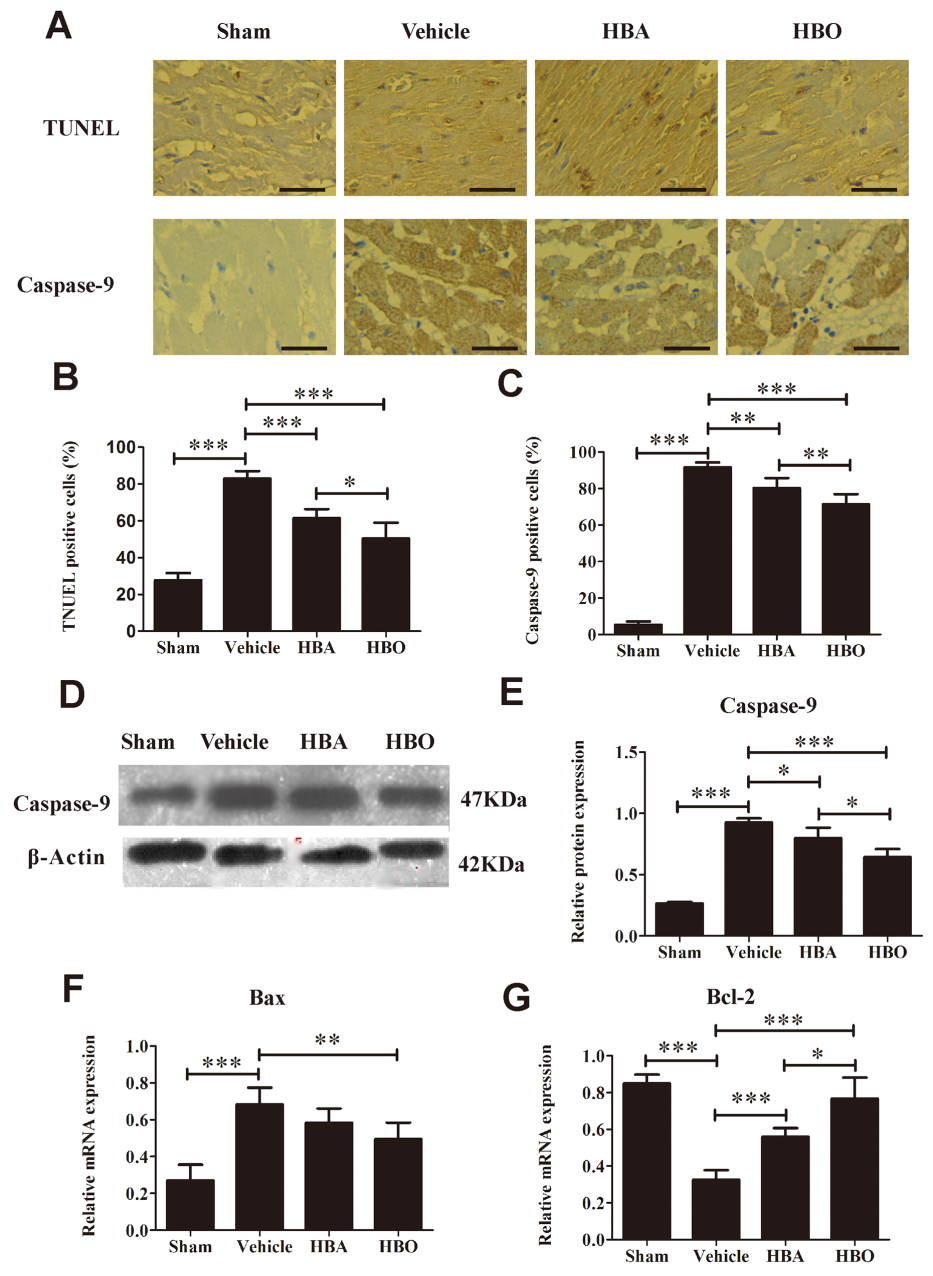
Evaluation of apoptosis in heart tissue **(A)** Upper row, representative images of TUNEL staining in various group. Lower row, representative images of immunohistochemical staining of caspase-9 myocardium. The scale bar represents 50 μm. **(B and C)** Quantitative analysis of TUNLE positive cells and caspase-9 positive cells. **(D and E)** Western blot assay of caspase-9 protein expression. **(F and G)** Bax and Bcl-2 mRNA expression determined by RT-PCR. The results are presented as the mean ± standard deviation (n = 5).^*^P < 0.05; ^**^P < 0.01; ^***^P < 0.001.

In this study, cardiomyocyte apoptosis was evaluated by terminal deoxynucleotide transferase dUTP nick end labeling (TUNEL) staining and caspase-9, Bax and Bcl-2 protein/gene expression. As shown in Figure 4A and 4B, the percentage of TUNEL-positive cells increased dramatically in the vehicle group versus the sham group. Compared with the vehicle group, the myocardial apoptotic index in the HBO group decreased. The result of the immunohistochemistry and western blot analyses of caspase-9 protein in the ischemic myocardial tissue of each group is shown in Figure 4A, 4C, 4D, and 4E. It was obvious that level of caspase-9 was higher in the vehicle group than in the sham group, whereas its expression was greatly decreased in the rats pretreated with HBO. The RT-PCR analysis (Figure 4F and 4G) demonstrated that the expression level of Bax mRNA was significantly increased, while Bcl-2 mRNA was significantly decreased in the vehicle group compared with the sham group. In contrast, HBO treatment significantly reversed the expression of Bax and Bcl-2 mRNA, whereas HBA treatment exhibited no significant difference compared with the vehicle group.

### HBO inhibited MIRI-induced autophagy dysfunction

**Figure 5:**
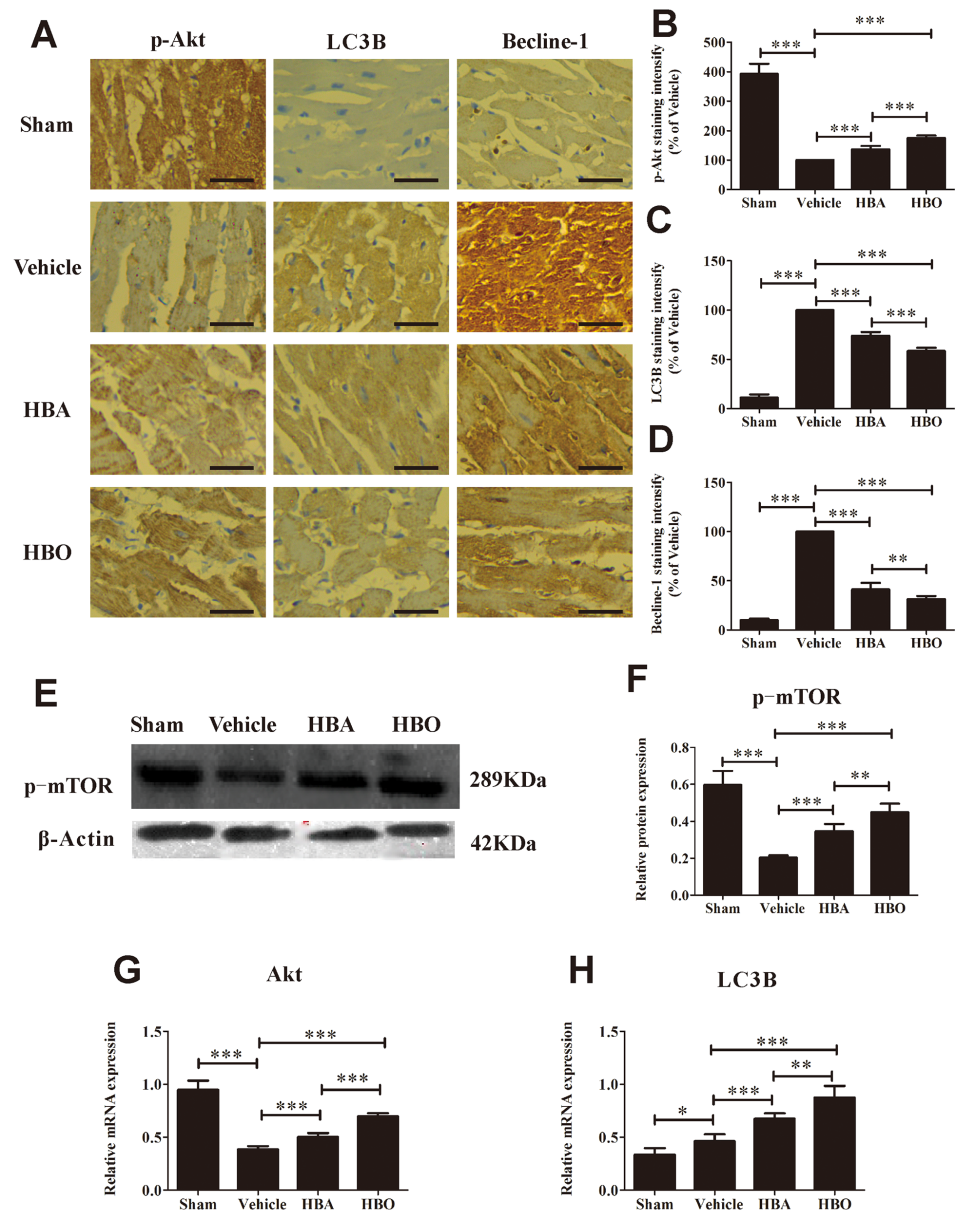
HBO inhibited MIRI-induced autophagy dysfunction **(A)** Immunohistochemical staining of p-Akt, LC3B, and Beclin-1 in heart tissue of each group. The scale bar represents 50 μm. **(B, C and D)** Quantitative densitometric analysis of p-Akt, LC3B, and Beclin-1 staining slides of rats subjected to MIRI. Data were normalized with vehicle and presented as percentage rates. **(E and F)** Western blot assay of p-mTOR protein expression. **(G and H)** Akt and LC3B mRNA expression determined by RT-PCR. The results are presented as the mean ± standard deviation (n = 5).^*^P < 0.05; ^**^P < 0.01; ^***^P < 0.001.

In addition to apoptosis, we investigated the effects of HBO on autophagy in the ischemic-reperfused myocardium. As shown in Figure 5A, 5B, 5C and 5D, the expression of p-Akt was decreased, while the expressions of LC3B and Beclin-1 were increased in the vehicle group compared with the sham group. Interestingly, the expression of p-Akt was up-regulated, and LC3B and Beclin-1 were down-regulated in the HBO group relative to the vehicle group. Next, we further explored the expression of autophagy-related genes or proteins. As expected, the expression of p-mTOR in the vehicle group was significantly lower than in the sham group and was increased by HBO (Figure 5E and 5F). The gene levels of Akt and LC3B, determined by RT-PCR, are shown in Figure 5G and 5H. MIRI significantly decreased Akt and increased LC3B gene expression compared with the sham group. Most importantly, the gene levels of Akt and LC3B were reversed by the HBO treatment.

## DISCUSSION

MIRI has a high morbidity and mortality worldwide, and solving this problem remains a considerable challenge. In our previous study [25], we confirmed that HBO preconditioning mitigated MIRI in rats, as manifested by an improvement of cardiac function, myocardial infarction area, and ATPase (Na^+^-K^+^-ATPase and Ca^2+^-Mg^2+^-ATPase) activity and decreased cardiac enzymes levels. In the present study, we use an MIRI model to demonstrate that HBO exerts a protective effect against MIRI, and the molecular mechanism involves the HBO-mediated inhibition of inflammation via TLR4-NF-κB and the modulation of autophagy via Akt-mTOR signaling. In addition, in accordance with our previous study [25], we also observed that the levels of caspase-9 and Bax decreased along with the increasing Bcl-2 by the HBO treatment, thereby preventing MIRI-induced apoptosis.

High myocardial specificity was required to detect the indexes of cTnI and cTnT and high sensitivity to determine the index of Mb in myocardial ischemic necrosis [26]. During myocardial ischemia, the integrity of the cardiac muscle cell membranes is damaged, causing myocardial enzymes and proteins, such as Mb, cTnI and cTnT, to be released into the peripheral blood. Our previous study demonstrated that HBO treatment decreased myocardial enzymes during MIRI. Consistent with our previous research, the results of this experiment showed that HBO decreased the serum Mb, cTnI and cTnT levels, and the data supports that HBO pretreatment improves myocardial injury in MIRI rats.

Inflammation, which releases inflammatory cytokines and causes neutrophil infiltration and aggregates cell injury, is now recognized as a key regulator of MIRI. Recent studies show that pro-inflammatory cytokines, including TNF-α, IL-1β, IL-6, IL-10, and ICAM-1, are overexpressed in heart tissue with ischemic-reperfusion injury [27, 28]. Therapeutic methods that down-regulate pro-inflammatory cytokine expressions effectively attenuate myocardial injury [28, 29]. Using a MIRI rat model established by ischemia for 30 min and reperfusion for 60 min, we observed that the levels of TNF-α, IL-1β, IL-6, IL-10, and ICAM-1 were significantly increased in the vehicle rat compared with the sham group. However, when the rats were pretreated with HBO for 14 days, the levels of the above-mentioned indexes were significantly decreased, which is consistent with a previous study in terms of TNF-α and IL-1β [30]. Since TLR4-NF-κB signaling mediates the inflammatory reaction and a timely interception of TLR4 might be critical to prevent adverse myocardial insult [31, 32], we wondered whether or not the action of HBO was mediated through the inhibition of this pathway. The degradation of inhibitory protein IκB presents as p-IκBα from the dimeric complex followed by the activation of NF-κB, which presents as p-NF-κB p65 [33, 34]. In this study, we found that ischemic-reperfusion significantly induced increased in the expression levels of TLR4, NF-κB, and p-IκBα. The activation of NF-κB was further evidenced by the degradation of IκB. Interestingly, preconditioning with HBO efficiently alleviated the expression of TLR4, NF-κB, and p-IκBα in the injured myocardium. These results suggested that the effect of HBO on inflammatory cytokine expression was mediated via the inhibition of the TLR4-NF-κB signaling pathway.

Cardiomyocyte apoptosis is a rare event in healthy myocardium but is the predominant form of cell death in infarcted myocardium. Studies show that apoptosis following MIRI is associated with the Bcl-2 family of proteins [35]. In opposition to Bax, Bcl-2 prevents the opening of the mitochodrial permeability transition pore (mPTP) to improve Ca ^2+^ overload, inhibit caspase activity, and thus reduce cell apoptosis [36]. Caspases, including caspase-3 and caspase-9, are critical enzymes in the execution of the apoptotic cascade pathway by the formation of apoptosis bodies [37]. The results showed that HBO preconditioning significantly increased Bcl-2 expression levels and decreased Bax mRNA expression with a corresponding decreased caspase-9 protein expression. Moreover, we used TUNEL assays to determine the extent of apoptosis of the cardiomyocytes of the different groups and found that TUNEL-positive cells were markedly increased in the vehicle group, whereas they were significantly decreased in the HBO-treated group. Our findings are consist with previous reports [29, 37], and indicate that HBO mediates its protective effect on MIRI by inhibiting myocardial apoptosis. In addition, p-Akt activates the downstream target eNOS, promotes the release of NO, and prevents the opening of mPTP, which thus inhibits apoptosis [38]. Data from our previous study demonstrate that pretreatment with HBO significantly increases the NO content and eNOS activity and, combined with present study finding that HBO preconditioning significantly increases p-Akt expression, suggests that the anti-apoptosis effect of HBO might be involved in the activation of the Akt-eNOS signal pathway.

Autophagy is a process of self-degradation of cellular components by which organelles or cytosol are breakdown [39]. It occurs constitutively as a housekeeping process and importantly involved in cardiovascular diseases such as MIRI [40, 41]. mTOR is a serine protein kinase that regulates cell growth, cell proliferation, and cell survival. As a major negative regulator of autophagy, it can be activate by PI3K/Akt, then negatively regulated the activity of autophage-initatation kinase ULK1 complex via its phosphorylation level [42, 43]. It has been reported that the regulation of the Akt-mTOR pathway provides cardioprotection by reducing autophagy and enhancing recovery in MIRI [44]. In this study, we observed that autophagy is excessively activated during MIRI, as characterized by the down-regulation of Akt and p-mTOR levels and the up-regulation of LC3B and Beclin-1 levels, whereas HBO treatment increased the expression of Akt and p-mTOR and decreased the expression of LC3B and Beclin-1. Beclin-1 participates in the process of the activation of autophagosomes to nucleation, which involves Atg complex. Since Atg proteins are recognized as the core molecular machinery of autophagy, we will explore Atg proteins and genes expression in our further experiment.

Moreover, recent studies have demonstrated that autophagy plays a crucial role in modulation of inflammation reaction, which involved in restricting the activation of inflammasomes [45, 46] and regulating the secretion of a number of cytokines [47], such as TNF-α and IL-1β. In our study, we observed that there was a reverse trend between autophagy and inflammation for the MIRI rats treated with HBO, which was consistent with the result from a previous study using transgenic mice with a cardiac-specific overexpression of mTOR [48].

In addition, since autophagy is demonstrated to engage the apoptotic pathway, it would be interesting to explore the relationship between them. Several studies reveal that autophagy may promote cell death via controlling apoptosis-regulatory proteins, such as p53, Bad, Bcl-2 and caspases [49, 50]. Our present study showed higher expressions of caspase-9, Bax, LC3B and Beclin-1 in the MIRI rats. However, the expression of these indexes was lower after HBO treatment, which supported that MIRI induces autophagy and apoptosis, and the inhibition of the autophagy decreases apoptosis. This phenomenon was also confirmed by rapamycin treatment, which is used as an mTOR inhibitor to induce autophagy [51]. Taken together, our findings suggested that the cardioprotective effect of HBO relates to the inhibition of autophagy via the mTOR signaling pathway. However, some limitations still exist in this study. First, the transmission electron microscopy has not been performed to observe the subcellular structure of cardiomyocytes. Secondly, the detailed mechanism of the activation and regulation of Atg are needed to clarify following the HBO treatment.

In conclusion, the present study showed that HBO efficiently prevented MIRI. The protective effects of HBO were primarily mediated through the inhibition of the inflammatory response and excessive autophagy via regulation of the TLR4-NF-κB signaling pathway and the mTOR signaling pathway, respectively. Hence, our results supported that HBO might be a useful agent for the prevention of MIRI in the clinic.

## MATERIALS AND METHODS

### Animals

The study was approved by our institutional ethical committee (Approval No.: 20110501202). Laboratory animal use and care were according to US guidelines (NIH publication No. 85-23, revised in 1996). Sprague-Dawley (SD) rats of both sexes (Grade SPF), weighing 180 - 220 g, were obtained from the Experiment Animal Center of Guangxi Medical University (Certificate No. SYXK 2009-0002). The rats were housed in a temperature-controlled room at 22 ± 2°C with a 12 h light and dark cycle and had access to food and water ad libitum.

### MIRI model and experimental protocol

After three days of acclimatization to laboratory conditions, 60 rats were randomly assigned to the following groups (n =15) by using random number table method: sham; vehicle, HBA and HBO. The surgical procedures were performed by occlusion of the LAD coronary artery and subsequent reperfusion according to a previous study [5]. Briefly, the rats were anesthetized, intubated and mechanically ventilated (Shanghai Alcott Biotech Co., Ltd). The chest was opened, and the heart was exposed. Next, the rats underwent a 30-min ligation (using a 5-0 silk suture) and 60-min reperfusion of the LAD coronary artery. The rats in the vehicle group received no intervention either before LAD occlusion or after reperfusion. Before surgical procedures, the rats in HBO group were placed in hyperbaric chambers (Yantai Hongyuan CO., Ltd) and received the HBO treatment once daily for 14 days, which was conditioned based on our previous reports [24]. The rats in HBA group received air treatment, in which the treatment procedure including the chamber, compression time, decompression time and treatment period were the same as the HBO group. The sham-operated rats were subjected to the same surgical procedure, but the ligation remained untied.

### Tissue preparation

At the end of reperfusion, the blood samples were collected before sacrificing the animals for biochemical assays. The heart was promptly removed, and the left ventricle was isolated. A number of the left ventricular samples were immersed in 10 % formalin, sectioned into 4 μm and prepared for TUNEL and immunohistochemical examination. Others were stored at -80°C for PCR and western blot analyses.

### Detection of myocardial enzyme levels in serum

To measure the heart muscle damage, some indicators, including cTnI, cTnT and Mb were evaluated in the serum. The levels of enzyme were determined using a 200FR NEO automatic biochemical analyzer (Toshiba, Japan).

### Determination of inflammatory cytokines in the serum

The concentrations of IL-1β, IL-6, IL-10, TNF-α, and ICAM-1 in the serum were determined using a SpectraMax Plus384 microplate reader (Molecular Devices, USA) according to the manufacturers’ instructions (Boster, China). A BCA kit was used to quantify the protein content.

### Assessment of cardiomyocyte apoptosis by TUNEL

The myocardial apoptosis was identified by the TUNEL assay using an in situ cell death detection kit according to the manufacturers’ protocol. The stained sections were observed under a microscope (Olympus, Germany). Five visual fields of each section were randomly selected for apoptotic cell counts. Cells with brown granules in the nuclei were determined as positive cells. The myocardial apoptotic index was expressed by the number of TUNEL-positive cardiomyocytes over the total number of cardiomyocytes (400×, Olympus, Germany).

### Immunohistochemical examination

Immunohistochemical procedures were conducted according to our previous study [52]. Briefly, the deparaffinized and hydrated slides were incubated in 3 % H_2_O_2_ for 30 min to block the endogenous peroxidase activity and were later placed in citrate buffer (PH = 6) for antigen retrieval. The specimens were incubated with the primary antibody overnight at 4°C (p-IκBα, 1: 100; LC3B, 1: 100; Beclin-1, 1: 100, Thermo Fisher Scientific, USA; p-NF-κB p65, 1: 100; caspase-9, 1: 50; p-Akt, 1: 100; and TLR4, 1: 100; Abcam, U.K). After three washes with 0.1 mol/L PBS, the sections were incubated with a biotinylated goat anti-rabbit immunoglobulin G secondary antibody (Zhongshan Goldenbridge Biological Technology, China) at 30°C for 25 min. Next, the specimens were incubated with a streptavidin-biotin complex at 30°C for 20 min and were further incubated with diaminobenzidine for 15 min at room temperature. The slides were counterstained with hematoxylin and were visualized under a microscope at a magnification of 400× (Olympus, Germany). The positive cell counts were analyzed in five independent sections and were captured using a pathological image analyzer (Leica DM6000, Germany). The immunopositive cell rate was calculated as the number of immunopositive cells divided by the total cells (including immunopositive cells and immunonegative cells).

### Western blot analysis

The left ventricular tissue was homogenized (12,000 rpm, 10 min, 4°C) with phosphate buffered saline and was incubated in lysis buffer. The supernatant was stored in a 0.5 ml centrifuge tube at -70°C, and 20 μg of protein was electrophoresed by sodium dodecyl sulfate polyacrylamide gel electrophoresis (SDS-PAGE) and was transferred to polyvinylidene fluoride (PVDF) membranes. The membranes were incubated in buffer with antibodies (p-IκBα, Thermo Fisher Scientific, USA; p-NF-κB p65, TLR4, caspase-9, and p-mTOR; Abcam, U.K) and then were incubated with the horseradish peroxidase secondary antibody (Zhongshan Goldenbridge Biological Technology, China). β-Actin was the protein loading control. The relative protein expression was quantified by densitometric scanning using Image Lab software (Bio-Rad Laboratories, Inc, USA) [53].

### Relative quantitative RT-PCR

The total RNA was extracted from ischemic myocardial tissue using the Trizol reagent (Invitrogen Life Technologies, USA). The total RNA was quantified by optical density measurement at 260/280 nm using a spectrophotometer (Thermo Scientific Company, USA). Reverse transcription was performed in a 20 μL reaction mixture containing 4 μg of total RNA. RNA was transcribed to cDNA with reverse transcriptase (Takara Bio, Japan). The reverse transcription products were stored at -80°C until use. The PCR primers of the target genes were synthesized by Sangon Biotechnologies (China, Table 1). Then, RT-PCR was performed using a cDNA template and by adding primer, mix and RNase-free water using a 7300 Real Time PCR system (Applied Biosystems, USA). The parallel amplification of rat β-Actin and GAPDH were performed for references. The levels of mRNA expression were determined using the 7300 Real Time PCR system SDS software according to the 2^-ΔΔCt^ method [54].

**Table 1:** The primer sequences of eight genes for real-time PCR

Gene	Forward primer (5′ – 3′)	Reverse primer (5′ – 3′)
IL-6	GAC GTT TCA GAG GTT CTC AGA G	TAG TCC TTC CTA CCC CAA TTT CC
TNF-α	CCC TCA CAC TCA GAT CAT CTT CT	GCT ACG ACG TGG GCT ACA G
NF-κB	GAA CGA TAA CCT TTG CAG GC	TTT CGA TTC CGC TAT GTG TG
TLR4	CAT GGA TCA GAA ACT CAG CAA AGT C	CAT GCC ATG CCT TGT CTT CA
Bax	CAG GAT GCG TCC ACC AAG AA	CGT GTC CAG GTC AGC AAT CA
Bcl-2	AGC GTC AAC AGG GAG ATG TC	TTC CAC AAA GGC ATC CCA GC
β-actin	AAC CCT AAG GCC AAC CGT CAA AAG	TCA TGA GGT AGT CTG TCA GGT
Akt	TGA GGT TGC CCA CAC GCT TA	GGC GTA CTC CAT GAC AAA GCA G
LC3B	GAT GTC CGA CTT ATT CGA GAG C	TTG AGC CTG TAA GCG CCT TCT A
GAPDH	GAC AAC TTT GGC ATC GTG GA	ATG CAG GGA TGA TGT TCT GG

### Statistical analysis

The values are expressed as the mean ± SE. The statistical analyses were performed using SPSS 13.0 software (SPSS Inc., USA). A one-way analysis of variance (ANOVA) was used to compare the data among the different groups, and the least significant difference (LSD) *t* test was carried out to compare the data between the groups. The level of significance was set at *P* < 0.05.
